# Contraception use and pregnancy among 15–24 year old South African women: a nationally representative cross-sectional survey

**DOI:** 10.1186/1741-7015-5-31

**Published:** 2007-10-28

**Authors:** Catherine MacPhail, Audrey E Pettifor, Sophie Pascoe, Helen V Rees

**Affiliations:** 1 Reproductive Health & HIV Research Unit, Department of Obstetrics and Gynecology, University of the Witwatersrand, Johannesburg, PO Box 18512, Hillbrow 2038, South Africa; 2 Department of Epidemiology, CB#7435 McGavran-Greenberg Building, University of North Carolina at Chapel Hill, Chapel Hill, NC 27599-7435, USA; 3 Infectious Disease Epidemiology Unit, Department of Infectious and Tropical Diseases, London School of Hygiene and Tropical Medicine, Keppel Street, London WC 1E 7HT, UK; 4 Reproductive Health & HIV Research Unit, Department of Obstetrics and Gynecology, University of the Witwatersrand, Johannesburg, PO Box 18512, Hillbrow 2038, South Africa

## Abstract

**Background:**

Adolescent reproductive health has not continued to receive the attention it deserves since the start of the HIV epidemic. In South Africa, high numbers of adolescent women report pregnancies that are unwanted and yet few have accessed available termination of pregnancy services. Enabling contraception use is vital for meeting the goals of HIV prevention.

**Methods:**

A nationally representative survey of South African 15–24 year olds was undertaken. Participants completed a questionnaire on sexual behaviour and provided an oral fluid sample for HIV testing. Analysis of the data was restricted to women (n = 6217), particularly those who reported being sexual active in the last 12 months (n = 3618) and was conducted using *svy *methods in the program STATA 8.0 to take account of sampling methods. Univariate and multivariate analyses were conducted to explore factors associated with contraceptive use.

**Results:**

Two thirds of all women reported having ever been sexually active and among these 87% were sexually active in the past 12 months. Among women who reported currently being sexually active, 52.2% reported using contraceptives. There was evidence of association between contraceptive use and being employed or a student (vs unemployed); fewer sex partners; type of last sex partner; having talked to last partner about condom use and having ever been pregnant.

**Conclusion:**

Specific emphasis must be placed on encouraging young women to use contraceptive methods that offer protection against pregnancy and STIs/HIV. Our consistent finding of a relationship between discussing condom use with partners and condom use indicates the importance of involvement of male partners in women's contraceptive decisions.

## Background

The two largest public health interventions directed at adolescents include the provision of contraceptive services and access to condoms for the prevention of HIV and other sexually transmitted diseases. Given the alarming prevalence of HIV, recent emphasis has been on HIV prevention to the detriment of the delivery of contraceptive services, despite estimates that 200 million women worldwide have unmet contraceptive needs [1]. Pregnancy rates remain high among young women [2,3] and termination of pregnancy is not always readily accessible, to ensure that women have the ability to prevent unwanted pregnancies. The provision of services that allow women to manage their reproductive health is key to women's health. Encouraging the use of contraceptive services is also important for meeting HIV prevention goals: it has been shown to be more cost effective to prevent the birth of HIV positive children through providing family planning to women in the general population than increasing the provision of Nevirapine for HIV-positive mothers within antenatal care [4]. Thus, contraception services should be recognised as contributing significantly to HIV prevention, in and of themselves [1].

Investing in family planning as a component of good reproductive health has benefits that go beyond the obvious prevention of pregnancy and reduction of disease burden. The social and economic benefits for global development goals should not be overlooked [5]. Failure to meet the reproductive health needs of young people is particularly prevalent among poor adolescents who are often marginalized from interventions located in mass media, schools and clinics [6]. In South Africa, where large numbers of young people live in conditions of poverty, such a lack of access to reproductive health services could translate into increased levels of unwanted pregnancy and disease among this group.

In this paper we describe the sexual behaviour of a nationally representative sample of young, sexually active South African women; in particular, their contraceptive use and prevalence of pregnancy. Specifically, we examine factors associated with contraception use through logistic regression so as to generate information that will assist in identifying strategies to encourage greater levels of contraception use among young women. Such information is particularly relevant for developing countries where population level data on contraception and levels of pregnancy among adolescent women is scarce.

## Methods

Data presented here were gathered through a nationally representative survey of 15–24 year olds living in the nine provinces of South Africa. The survey employed a three-stage disproportionate, stratified design using the 2001 national census as the sampling frame. The primary sampling units were census enumeration areas (EAs) and included both rural and urban communities. Thereafter, selected households were enumerated and within households with eligible youth, a single eligible youth was randomly selected to participate in an interview and to provide a sample for HIV testing. Additional information on the methods employed in this survey are available elsewhere [7,8]. A total of 11904 youth participated in the survey; however, these analyses are limited to the young women in the sample who reported being sexually experienced (n = 4066).

### Interview

A comprehensive, structured questionnaire was administered to participants by trained interviewers. The questionnaire elicited information on demographics, sexual history and behaviours, attitudes and norms relating to HIV and exposure to a national HIV prevention programme for South African youth. The questionnaire was translated from English into eight local South African languages and back translated to ensure correctness of meaning and interpretation. Analyses in this paper made use of questions about history of pregnancy, current contraceptive use among sexually active women, condom use at last sex, and consistency of condom use with last partner.

After completing the questionnaire, an oral fluid specimen was collected from each participant using the Orasure^® ^HIV-1 Oral Specimen Collection Device (Orasure Technologies Inc., Bethlehem, PA, USA). Orasure^® ^samples were centrifuged and the eluted sample tested for HIV-1/2 antibodies using the Vironostika Uni-Form II HIV-1/2 plus O MicroElisa System (Biomerieux, Durham, NC, USA) at a central Johannesburg laboratory.

Informed consent was obtained from all young people and from the parents of youth aged 15–17 years. The study was approved by the Committee for the Protection of Human Subjects, University of the Witwatersrand, South Africa.

### Description of measures

Women who reported having ever having had vaginal or anal sex were asked whether they were currently using a method of contraception. Those who answered 'yes' were then asked "What methods are you currently using?". Different contraceptive options were not read out to participants and women were allowed to report using more than one method. To determine pregnancy history, female respondents who reported having ever been sexually active were asked "Have you ever been pregnant?" and were also asked ''Are you currently pregnant?"; women were classified as having ever been pregnant if they answered affirmatively to either question. Women who reported having ever been pregnant were asked "Have you ever been pregnant when you didn't want to be?" and thereafter were asked "Have you ever terminated (aborted) a pregnancy?".

### Statistical analyses

The final sample was weighted to account for differential sampling probabilities, and to represent the distribution of young people aged 15–24 years based on the 2001 South African census. This analysis was restricted to those women who had been sexually active, specifically within the last 12 months (n = 3618). Analyses were conducted using STATA 8.0 (College Station, TX, USA). Statistical testing was conducted using *svy *methods allowing for sample strata, primary sampling units and population weights. Factors associated with contraceptive use were examined using univariate and multivariate analyses. Categorical data were analysed using chi-square tests, with multivariate analysis by logistic regression.

Possible predictors of contraceptive use that were significant at the 90% level on univariate analysis (p < 0.1), and those variables where existing knowledge provided evidence for inclusion, were each included in a multivariate model adjusted for *a priori *confounders (age group, education, marital status, current employment status and race). Where appropriate, non-dichotomous variables were collapsed to dichotomous categories in order to increase the power of the model. A backwards elimination process was used to identify those variables that were not independently associated with contraceptive use after adjustment. These variables were dropped from the model, keeping in variables that had been selected based on *a priori *hypotheses, and the final parsimonious model presented.

## Results

The total sample of young women included in this study numbered 6217. Of this number, 67.9% (n = 4066) reported ever having had sex. Slightly more than half of sexually experienced women (52.2%) reported currently using contraception. Amongst those women who reported that they were currently using a contraceptive 26.5% (95% CI: 20.9–33.1%) were using condoms only (dual protection); 6.8% (95% CI: 4.4–10.4%) were using dual method (barrier and hormonal); and 66.6% (95% CI: 57.5–74.7%) were using only a hormonal method of contraception. A small percentage of women reported using less reliable methods (1.1% of contraceptive users) such as natural, rhythm, withdrawal or safe period. These women were excluded from later analysis. Half of sexually experienced women reported having ever been pregnant (95% CI: 46.4–52.7%), of whom 65% indicated that their pregnancy had been unwanted. Despite this, only 2.6% reported having accessed termination of pregnancy. In terms of contraceptive method use, there were strong differences by pregnancy status; women who reported ever being pregnant were much more likely to report using hormonal methods and less likely to use male condoms compared to women who had never been pregnant (81.8% vs 47.0%, p < 0.001; 9.8% vs 47.6%, p < 0.001, respectively). The final analytic sample was restricted to women sexually active in the past 12 months (n = 3618).

### Women sexually active in the past 12 months

Among sexually experienced women 87% (n = 3618) reported having had sex in the past 12 months. HIV prevalence in this population was 19.7% (95% CI: 16.6–23.2%). The vast majority of these women reported that their last partner was a main partner (98.5%) and that they had been with this partner for a month or more (87%). A total of 44% reported condom use at last sex.

### Characteristics of women sexually active in the past 12 months reporting contraception use

In univariate analysis (see Table 1), women who were employed or still a student were 1.7 (95% CI: 1.2–2.3) and 1.6 (95% CI: 1.2–2.2) times as likely, respectively to use a contraceptive than those that were unemployed (p < 0.001). There was some evidence that women who had completed their high school education or were still currently in school (59%) were also more likely (OR = 1.3; 95% CI: 1.0–1.6) to be currently using contraceptives than women who were no longer in school and had not completed their high school education (53%) (p = 0.071).

**Table 1:** Contraceptive use amongst woman who have been sexually active within the last 12 months (n = 3618)

	**Characteristic**	**No. using contraceptive (weighted %)**		**p Value**	**OR**
Geotype	Rural formal	197/316	(60.5%)		1.0
	Rural informal	782/1 503	(56.3%)		0.8 (0.4–1.6)
	Urban formal	932/1 586	(56.2%)		0.8 (0.5–1.3)
	Urban informal	124/213	(57.1%)	p = 0.907	0.9 (0.5–1.6)
Electricity to house	No	402/781	(55.7%)		1.0
	Yes	1627/2 827	(57.0%)	p = 0.891	0.9 (0.5–2.0)
Age (years)	15–19	849/1 566	(59.0%)		1.0
	20–24	11186/2 052	(55.2%)	p = 0.421	0.9 (0.6–1.2)
Education	Not completed high school	792/1 436	(53.2%)		1.0
	In school/completed high school	1242/2 180	(59.1%)	p = 0.071	1.3 (1.0–1.6)
Marital status	Single	1929/3 421	(56.7%)		1.0
	Married	105/196	(55.1%)	p = 0.786	0.9 (0.6–1.5)
Occupational status	Unemployed	879/1 670	(50.6%)		1.0
	Employed	272/434	(63.1%)		1.7 (1.2–2.3)
	Student	882/1 511	(62.3%)	p < 0.001	1.6 (1.2–2.2)
Race	White/Coloured/Indian	237/416	(51.7%)		1.0
	Black	1798/3 202	(57.2%)	p = 0.284	1.2 (0.8–1.9)
HIV status	Not infected	1629/2 912	(57.2%)		1.0
	Infected	406/706	(54.4%)	p = 0.518	0.9 (0.6–1.3)
No. of lifetime partners	1 lifetime partner only	855/1 487	(59.1%)		1.0
	> 1 lifetime partners	1178/2 126	(54.8%)	p = 0.163	0.8 (0.7–1.1)
No. of partners in last 12 months	1 partner only	1790/3 155	(57.9%)		1.0
	More that one partner	245/463	(47.7%)	p = 0.003	0.7 (0.5–0.9)
Sexual debut	15 or younger	1572/2 753	(55.2%)		1.0
	Over 15 years	462/863	(61.5%)	p = 0.454	1.3 (0.7–2.6)
Type of last partner	Main partner	2000/3 541	(57.0%)		1.0
	Casual partner	27/65	(30.2%)	p < 0.001	0.3 (0.2–0.6)
Length of last relationship	More than one month	1769/3 081	(56.8%)		1.0
	1 month or less	221/458	(58.0%)	p = 0.873	1.0 (0.6–1.9)
Age difference to last partner	Less than 10 years	1902/3 353	(57.1%)		1.0
	10 years or more	108/220	(49.6%)	p = 0.155	0.7 (0.5–1.1)
Freq of sex in past month	None	629/1 304	(51.7%)		1.0
	1–5 times	1151/1 892	(60.1%)		1.4 (1.0–2.1)
	More than five times	228/375	(58.9%)	p = 0.074	1.4 (0.8–2.5)
Talked to last partner about using condoms	No	376/801	(41.2%)		1.0
	Yes	1651/2 802	(60.7%)	p < 0.001	2.2 (1.5–3.3)
Ever been pregnant	No	951/1 834	(52.7%)		1.0
	Yes	1084/1 782	(60.6%)	p = 0.002	1.4 (1.1–1.7)
Ever had unwanted pregnancy	No	369/601	(62.8%)		1.0
	Yes	713/1 179	(59.4%)	p = 0.327	0.9 (0.7–1.2)
Parents know where you are at night	No – not always	662/1 255	(54.0%)		1.0
	Yes	978/1 681	(58.4%)	p = 0.140	1.2 (0.9–1.5)
How strict are the rules at home	Not strict/not very strict	602/1 084	(52.5%)		1.0
	Strict/very strict	1044/1 871	(58.5%)	p = 0.061	1.3 (1.0–1.6)
Can approach parents/guardians about sex	No/not sure	897/1 645	(53.4%)		1.0
	Yes	1137/1 971	(59.2%)	p = 0.109	1.3 (0.9–1.7)
Parent/guardian taking care of them at home	Yes	1843/3 272	(55.8%)		1.0
	No	191/345	(63.5%)	p = 0.076	1.4 (1.0–2.0)
Condom accessibility	Very/somewhat easy	1936/3 430	(57.2%)		1.0
	Very/somewhat difficult/not sure	95/182	(44.9%)	p = 0.105	0.6 (0.3–1.1)

Interestingly, those women who had more than one sexual partner in the past 12 months appeared to be less likely to be currently using contraception than women who had had only one partner in the past 12 months (OR = 0.7; 95% CI: 0.5–0.9). Those governed by strict rules at home were 1.3 times more likely (95% CI: 1.0–1.6) to be using contraceptives than those who were not governed by strict rules (p = 0.061).

There is no evidence of association between those women who had an early sexual debut (15 or younger) and those using contraception (OR = 1.3; 95% CI: 0.7–2.6), neither was there significant evidence of an association between those who have had their sexual debut within the last 2 years and current contraception use (OR = 0.9; 95% CI: 0.6–1.3) [data not shown]. The length of last relationship was also not associated with contraceptive use; those women whose last relationship was more than a month were no more likely to use contraceptives than women whose last relationship lasted less than a month (OR = 1.0; 95% CI: 0.6–1.9; p = 0.873). Women whose last relationship was with their main partner, however, were more than three times more likely than those women whose last partner was a casual partner to be currently using contraceptives (OR = 3.1; 95% CI: 1.6–5.8). There was no difference in contraceptive use by whether women reported perceiving themselves to be at risk of HIV or by self-reported symptoms of sexually transmitted infections.

As might be expected, women who had had sex with their last partner within the last month were more likely to be using contraceptives than those women who had not had sex recently (OR = 1.4; 95% CI: 1.0–2.1; p = 0.072). Women who reported having talked to their partner about condom use are more than twice as likely (OR = 2.2; 95% CI: 1.5–3.3) to be currently using contraceptives as women who did not talk to their last partner about using a condom. There was some evidence that women with a parent/guardian at home and who could discuss sex with their guardians/parents were more likely to use contraceptives (OR = 1.4; 95% CI 1.0–2.0, p = 0.076 and OR = 1.3; 95% CI: 0.9–1.7; p = 0.109 respectively); and that those who thought it was difficult to access condoms were less likely to use contraceptives (OR = 0.6; 95% CI: 0.3–1.1; p = 0.105).

Contraceptive use was associated with having ever been pregnant (OR = 1.4; 95% CI: 1.1–1.7), but is not associated with having had an unwanted pregnancy (OR = 0.9; 95% CI 0.7–1.2), having been pregnant before the age of 18 (p = 0.836) or the number of pregnancies (p = 0.366) (data not shown).

### Multivariate analysis of contraception use among women sexually active in the past 12 months

In multivariate analysis, after adjusting for age group, education, marital status, current employment status and race, and dropping variables not independently associated with contraceptive use after adjustment (strictness of rules at home; condom accessibility and having a parent/guardian to take care of them) the following variables remained in our parsimonious model. In terms of sexual behaviour, number of partners in the past 12 months remained independently associated with contraception use (OR = 0.7; 95% CI: 0.5–0.9), as did type of last partner (OR = 0.4 95% CI: 0.2–0.9); reporting sex in the past month (OR = 1.5; 95% CI: 1.1–2.0) and having talked to last partner about condoms (OR = 2.2; 95% CI: 1.5–3.3). Contraception use continued to be associated with being employed or a student (OR = 1.8; 95% CI: 1.3–2.6 and OR = 1.9; 95% CI: 1.3–2.7 respectively) and with having ever been pregnant (OR = 1.9; 95% CI: 1.5–2.5). In all cases there was little evidence of confounding by any of the *a priori *factors or predictors of contraceptive use within the multivariate model (see Table 2). Marital status was also dropped from the model due to its co-linearity with type of last partner (all married women who reported sex in the last 12 months reported that their partner was their main partner).

**Table 2:** Predictors of contraceptive use in women who have been sexually active in the last 12 months

	**Characteristic**	**OR**	AOR*
Age (years)	15–19	1.0	1.0
	20–24	0.9 (0.6–1.2)	0.8 (0.6–1.2)
Education	Not completed high school	1.0	1.0
	In school/completed high school	1.3 (1.0–1.6)	1.0 (0.8–1.3)
Occupational status	Unemployed	1.0	1.0
	Employed	1.7 (1.2–2.3)	1.8 (1.3–2.6)
	Student	1.6 (1.2–2.2)	1.9 (1.3–2.7)
Race	White/Coloured/Indian	1.0	1.0
	Black	1.2 (0.8–1.9)	1.4 (0.9–2.0)
No. of partners in last 12 months	One partner only	1.0	1.0
	More than one partner	0.7 (0.5–0.9)	0.7 (0.5–0.9)
Type of last partner	Main partner	1.0	1.0
	Casual partner	0.3 (0.2–0.6)	0.4 (0.2–0.9)
Sex in past month	No	1.0	1.0
	Yes	1.4 (1.0–2.1)	1.5 (1.1–2.0)
Talked to last partner about using condoms	No	1.0	1.0
	Yes	2.2 (1.5–3.3)	2.2 (1.5–3.3)
Ever been pregnant	No	1.0	1.0
	Yes	1.4 (1.1–1.7)	1.9 (1.5–2.5)

*AOR – all variables adjusted for age, education, occupational status and race as *a priori *confounders.

## Discussion

The results of this study show that by age 24 years, over two thirds of young South African women report being sexually experienced and 50% have been pregnant, yet only half report using contraception. In a context where HIV prevalence increases rapidly from 4.1% among 15 year old to 26.3% among 24 year old women [8], high levels of sexual activity and unprotected sex are placing these young women at risk of HIV infection, as well as pregnancy. International studies have highlighted the negative consequences of pregnancy for adolescent women, their babies and extended families [9,10]. The South African literature is not, however, as definite about such negative consequences. While Varga [11] and Cunningham and Boult [12] note that teenage pregnancy compromises education and future social and financial security, other authors have intimated that the changing social characteristics of South African society have diminished the social harms of teenage pregnancy [2,13,14]. Our research has previously indicated an association between HIV and limited education and unemployment among young women [7]. Our results, therefore, indicate that teenage pregnancy among South African women might be the precursor for outcomes that are later associated with HIV infection, such as school drop out [15]. Despite HIV being a more manageable chronic condition with the availability of ARVs, the disease continues to have significant physical, psychological and social consequences [16-19].

The data here indicate that contraceptive use among South African adolescent women is significantly associated with both employment and educational status. Changes in the South African education system mean that more young people are accessing primary education but unemployment remains a concern for young men and women. Interestingly, women were more likely to use contraception when reporting a single partner in the last 12 months, when reporting a main partner and when reporting increased sexual activity in the past month. This indicates that young women are considering the use of contraceptives only once they are involved in long-term, regular relationships. There remains, however, a need to offer contraceptive services to young women who are intermittently sexually active in less stable types of relationships.

Among young South African women, contraceptive use was associated with having previously been pregnant. In particular, we note that women who had ever been pregnant were more likely to report using hormonal methods and less likely to report using condoms compared to those young women who had never been pregnant. International studies have indicated that a lack of contraceptive use is often the result of social stigma or lack of knowledge. It is only after a first pregnancy that young women are educated about and subsequently offered contraceptive services, with preference being given to hormonal methods. This preference of health care providers to offer hormonal methods to young mothers is also true for young women in long-term relationships [20,21]. Again, this indicates the need for greater promotion of condoms as dual protection or inclusion of condoms as dual method use so as to add protection against disease to pregnancy prevention.

Our data indicate that the majority of women choose to use hormonal methods of contraception rather than barrier methods and that young women make contraceptive trade-offs in which condom use declines as relationship length increases [22]. Our previous research among South African adolescents confirms that the issue of trust becomes a significant factor in contraceptive decision making in longer term relationships, usually to the detriment of condom use [23]. Among women in long-term stable relationships there appears to be relatively limited use of contraceptive methods that offer protection from sexually transmitted infections and HIV infection. Family planning programmes for young women need to invest time and effort in encouraging young women to think of prevention in terms of both pregnancy and disease. Particular emphasis needs to be placed on empowering young women in long-term relationships to maintain their use of STI and HIV preventing methods.

It is significant that one of the factors associated with contraceptive use among this population is reporting having discussed condoms with last partner. We have also found this relationship with condom use at last sex [24] and other researchers have noted that limited contraceptive discussions between partners are associated with inconsistent use [25]. This suggests that part of the solution to limited contraceptive use lies in involving male partners and working with young people on developing their communication skills in sexual relationships. The current location of family planning services as distinct from generic health care services provides little opportunity for young men to be involved in their partners' contraceptive decision making.

Among the young women in this sample a large number reported their pregnancies as 'unwanted' yet few had accessed and utilised termination of pregnancy services, despite abortion being legally available in South Africa. These low reported levels of access to termination of pregnancy services are unusual; other African countries report high rates of abortion among adolescent women [26,27]. Various authors report that failure to use termination of pregnancy services by South African adolescents is due to lack of knowledge or social stigma [28,29]; and that teenage use of these services has been declining since their introduction in 1997 [30]. It is likely that the rates reported in our data are the result of underreporting of pregnancy termination given predominately negative adolescent views of abortion. In a study in one South African province, Varga noted that adolescents viewed the termination of pregnancies as a sin, socially irresponsible and indicative of a lack of moral strength [28]. Although young women do access abortions, she concluded that fewer terminations were occurring than were desired because of failed home abortions and procrastination until the pregnancy was too advanced for termination. Additionally, lack of knowledge about the legal status of pregnancy termination [29] uneven distribution of services [31] and poor provider attitudes towards providing termination of pregnancy [32] mean that many young women continue to have 'backstreet' abortions that are undocumented and not openly discussed.

The same behaviours are at the root of adolescent pregnancy and HIV infection, yet there is disagreement in the literature as to whether prevention programmes should integrate HIV and pregnancy prevention or not [33]. Stein [34] has noted that family planning, sexually transmitted infection and HIV prevention, have traditionally been located as separate services. In most instances, they have failed to accommodate the changing sexual and reproductive health needs of their clients by not becoming more integrated and not adequately recognising the necessity of referral between the different components. Research among South African adolescents has indicated that young people themselves are increasingly making the link between pregnancy and HIV. Rutenberg et al. [35] highlighted that among some adolescents the danger of HIV infection has become part of the risk-benefit equation when thinking about pregnancy. It is, therefore, clear that the opportunity exists for the integration of services and that there is already demonstrated acceptability of such integration among adolescent women.

## Conclusion

Levels of adolescent pregnancy and HIV infection are high in South Africa and it is thus imperative that equal weight is given to ensuring that young people have the skills and the resources to prevent infections and pregnancy and that there is an environment that is supportive of them doing so. South Africa has the best provision of reproductive health care and HIV prevention programmes in sub-Saharan Africa, yet the data presented here indicate that we continue to fail young women in terms of ensuring their access to and use of contraceptives; to termination of pregnancy services; and to HIV prevention. Our data indicate that beyond unprotected sex as the root cause of pregnancies and HIV infections, those behaviours that are likely to result in teenage pregnancy are also those that place young women at greater risk of HIV infection. Now, more than ever, there is a need to continue efforts to better integrate family planning and HIV prevention services and to involve young males in these decision making processes. Although efforts are being made to better integrate these different services, our efforts thus far have not been adequate.

## Competing interests

The author(s) declare that they have no competing interests.

## Authors' contributions

CM conceptualized and wrote the paper and was involved in the interpretation of results. AP designed the survey and data collection tools, managed the data collection process and was involved in the interpretation of results and development of intellectual content. SP conducted the statistical analysis presented in the paper and provided assistance in discussion of intellectual content. HR was the principal investigator of the study and was involved in the design of the survey and the data collection tools used. She was also involved discussion of the intellectual content of the manuscript. All authors read and approved the final manuscript.

## Pre-publication history

The pre-publication history for this paper can be accessed here:


